# Conservation of Small Regulatory RNAs in *Vibrio parahaemolyticus*: Possible role of RNA-OUT Encoded by the Pathogenicity Island (VPaI-7) of Pandemic Strains

**DOI:** 10.3390/ijms20112827

**Published:** 2019-06-10

**Authors:** Nicolás Plaza, Diliana Pérez-Reytor, Sebastián Ramírez-Araya, Alequis Pavón, Gino Corsini, David E. Loyola, Víctor Jaña, Leonardo Pavéz, Paola Navarrete, Roberto Bastías, Daniel Castillo, Katherine García

**Affiliations:** 1 Instituto de Ciencias Biomédicas, Facultad de Ciencias de la Salud, Universidad Autónoma de Chile, Santiago 8320000, Chile; nicolas.plaza@uautonoma.cl (N.P.); diliana.perezr@gmail.com (D.P.-R.); seba264@gmail.com (S.R.-A.); a.pavon@uautonoma.cl (A.P.); gino.corsini@uautonoma.cl (G.C.); dloyolac@gmail.com (D.E.L.); 2 Departamento Ciencias Básicas, Facultad de Ciencias, Universidad Santo Tomás, Santiago 8320000, Chile; 3 Facultad de Medicina Veterinaria y Agronomía, Universidad de las Américas, Sede Providencia, Santiago 8320000, Chile; victor.jgaray@gmail.com; 4 Instituto de Ciencias Naturales, Universidad de Las Américas, Santiago 8320000, Chile; lpavez@udla.cl; 5 Departamento de Ciencias Químicas y Biológicas, Universidad Bernardo O’Higgins, Santiago 8320000, Chile; 6 Laboratorio de Microbiología y Probióticos, Millenium nucleus in the Biology of Intestinal Microbiota, INTA, Universidad de Chile, Santiago 8320000, Chile; pnavarre@inta.uchile.cl; 7 Laboratorio de Microbiología, Instituto de Biología, Pontificia Universidad Católica de Valparaíso, Valparaíso 2340000, Chile; roberto.bastias@pucv.cl; 8 Marine Biological Section, University of Copenhagen, Strandpromenaden 5, DK-3000, 1353 Helsingør, Denmark; danielcastillobq@gmail.com

**Keywords:** *Vibrio parahaemolyticus*, sRNA, svpa1401.1, svpa1453.1, RNA-OUT, transposase, VPA1379, IS10, antisense

## Abstract

Small regulatory RNAs (sRNAs) are molecules that play an important role in the regulation of gene expression. sRNAs in bacteria can affect important processes, such as metabolism and virulence. Previous studies showed a significant role of sRNAs in the *Vibrio* species, but knowledge about *Vibrio parahaemolyticus* is limited. Here, we examined the conservation of sRNAs between *V. parahaemolyticus* and other human *Vibrio* species, in addition to investigating the conservation between *V. parahaemolyticus* strains differing in pandemic origin. Our results showed that only 7% of sRNAs were conserved between *V. parahaemolyticus* and other species, but 88% of sRNAs were highly conserved within species. Nonetheless, two sRNAs coding to RNA-OUT, a component of the Tn10/IS10 system, were exclusively present in pandemic strains. Subsequent analysis showed that both RNA-OUT were located in pathogenicity island-7 and would interact with transposase VPA1379, according to the model of pairing of IS10-encoded antisense RNAs. According to the location of RNA-OUT/VPA1379, we also investigated if they were expressed during infection. We observed that the transcriptional level of VPA1379 was significantly increased, while RNA-OUT was decreased at three hours post-infection. We suggest that IS10 transcription increases in pandemic strains during infection, probably to favor IS10 transposition and improve their fitness when they are facing adverse conditions.

## 1. Introduction

Small regulatory RNAs (sRNAs) are short (~18 to 30 nucleotides), noncoding RNA molecules [1]. Many studies on bacterial processes established that sRNAs mainly modulate their target gene expression post-transcriptionally [2]. These molecules can inhibit translation and decrease the expression of a target gene, but they can also activate gene expression [3]. The main control mechanisms of sRNAs that regulate genes are the modulation of protein activity, *trans*-acting, *cis*-acting, 5′ UTR elements, and the CRISPR–Cas system [4]. sRNAs acting in *cis* are coded in the opposite strand of their target mRNA with perfect complementarity, while sRNAs acting in *trans* interact with genes on separate loci, and union with the target gene depends on the binding to the Hfq protein [5].

The sRNAs play an important role in the regulation of pathogenesis and adaptation to the environment in the *Vibrionaceae* family [6,7], which includes important human pathogens such as *Vibrio cholerae* and *Vibrio parahaemolyticus,* mainly related to gastroenteritis cases, and *Vibrio vulnificus,* associated with extraintestinal infections [8,9]. Many studies have been carried out on *V. cholerae*, where the role of several sRNAs is well-known. Some examples are the role of sRNA Vrra in the control of outer membrane porins [10], the regulation of a colonization factor, the RbmC attachment factor [11], and the role of TarA (Tar: ToxT-activated RNA) and TarB [12], both involved in the regulation of virulence genes [13]. However, few studies have been performed on sRNAs in *V. parahaemolyticus* [4] and, consequently, knowledge about *V. parahaemolyticus* is scarce.

*V. parahaemolyticus* is an important global pathogen associated with foodborne infections. Gastroenteritis associated with seafood consumption drastically increased in Chile when pandemic strain O3:K6 reached the southern coast, and decreased after this strain disappeared [14]. However, other nonpandemic *V. parahaemolyticus* strains are currently responsible for clinical cases, including strains lacking classical virulence factors such as thermostable direct hemolysin (*tdh)*, *tdh*-related hemolysin (*trh),* and the Type III secretion system of chromosome 2 (T3SS2) [15]. Thus, the question arises if some sRNAs could have a role in the pathogenesis of *V. parahaemolyticus,* especially since this process has been suggested to be multifactorial [16].

Although it is accepted that the conservation of genes across species is a good indicator of similar functions [17], to date, the extent of sequence conservation of sRNAs in *V. parahaemolyticus* is unknown. Only two of the 43 sRNAs described in *V. parahaemolyticus* have been experimentally studied, RyhB [18] and Spot42 [19]. The former modulates the expression of several genes related to motility, chemotaxis, biofilm formation, and iron metabolism [18,20], while Spot42 post-transcriptionally regulates the expression of chaperone protein VP1682 of the Type III secretion system of chromosome 1 (T3SS1) [19], suggesting a role in cytotoxicity. Another study recently showed that, besides Spot42 and RhyB, one *qrr* sRNA was significantly upregulated during infection of pandemic *V. parahaemolyticus* in an animal model compared to laboratory culture conditions [21]. Similarly, our group also demonstrated that the expression of sRNAs in *V. parahaemolyticus* changes when bacterial growth conditions are modified [20].

Given the importance of sRNAs in the regulation of many processes in other *Vibrio* species, especially in *V. cholerae*, the lack of knowledge on sRNAs in *V. parahaemolyticus* could mean the lack of a fundamental element to understand important processes such as pathogenesis, metabolism, and/or responses to environmental stress. In this exploratory study, we determined the conservation of sRNAs between *V. parahaemolyticus* and other human pathogenic *Vibrio* species, but also between Chilean strains differing in pandemic origin. Our results suggested that RNA-OUT, exclusively present in pandemic strains, would regulate the expression of VPA1379, a transposase coded into pathogenicity island seven (VPaI-7), fitting the model for pairing IS10-encoded antisense RNAs. Additionally, our results suggest that IS10 transcription increases during infection, probably to favor IS10 transposition, and, consequently, to improve the fitness of pandemic strains under stress conditions.

## 2. Results

### 2.1. Gene Conservation across Human Pathogenic Vibrio Species

**Figure 1:**
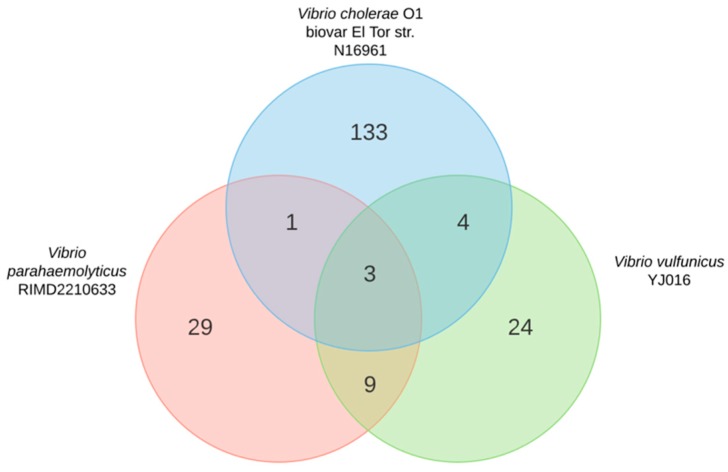
Venn diagram of small regulatory RNAs (sRNAs) reported for three human pathogenic *Vibrio* species in the Bacterial Small RNA Database.

If gene conservation across species is a good indicator of similar functions, we would expect that sRNAs conserved between *V. parahaemolyticus* and other human pathogenic species, such as *V. cholerae* and/or *V. vulnificus,* to fulfill the same or a similar function. However, our results showed that the conservation of sRNAs between human pathogenic *Vibrio* species was low. When sRNA sequences were clustered using 90% identity, only three sRNAs (RNaseP_bact_a, Spot42, and RyhB) were conserved among all strains (Figure 1). Comparison between pairs of species showed that only a mini-ykkC was shared between *V. parahaemolyticus* and *V. cholerae*, while nine sRNAs were shared with *V. vulnificus*: S15, Qrr (svpa1316.1), Qrr (svpa783.1), ffs, P26, GcvB, 6S RNA, FMN riboswitch, and Thr_leader (Figure 1), four of which are classical highly conserved sRNAs in bacteria (S15, ffs, P26, and 6S RNA).

### 2.2. Small RNA Conservation and Distribution in Strains Differing in Pandemic Origin

**Figure 2:**
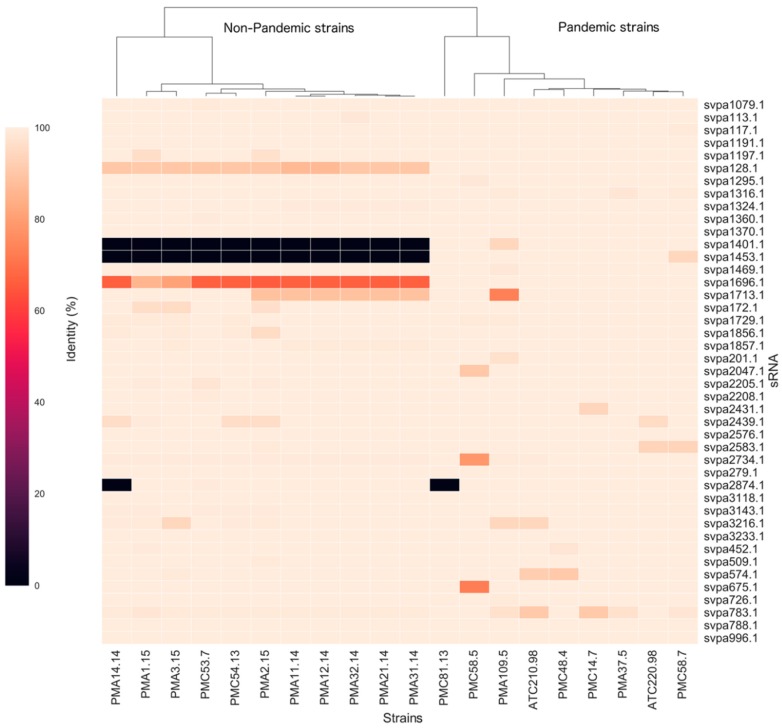
Percentage heat map of sRNA identity sequences showing differences between *V. parahaemolyticus* strains compared to sRNA sequences reported for reference strain RIMD2210633. Color gradient represents 0% (black) to 100% (skin color) of identity in twenty strains. The dendrogram was constructed using the Euclidean method to cluster the main groups.

A total of 43 sRNAs described in the Bacterial Small RNA Database (BSRD) for the pandemic *V. parahaemolyticus* RIMD2210633 (Table S1) were identified in 20 Chilean strains of *V. parahaemolyticus* (Table 1). Nine of them, namely, svpa279.1 (Alpha_RBS), svpa788.1 (mini-ykkC), svpa996.1 (ffs), svpa1079.1 (Cyclic di-GMP-II riboswitch), svpa1191.1 (Lysine riboswitch), svpa1370.1 (Purine riboswitch), svpa2576.1 (S15), svpa3118.1 (P26), and svpa3233.1 (thiamin pyrophosphate (TPP) riboswitch), were highly conserved between pandemic and nonpandemic strains, with 100% identity compared to the sRNAs described in reference strain RIMD2210633, of pandemic origin (Figure 2, Table S2). Interestingly, svpa117.1 (Spot42) and svpa113.1 (RyhB), the two most studied sRNAs in *V. parahaemolyticus*, also showed high-percentage identity between pandemic and nonpandemic strains compared to the reference sequences of RIMD2210633 (Figure 2, Table S2).

However, svpa128.1 (TPP riboswitch), svpa1713.1 (RyeB), and svpa1696.1 (Cyclic-di-GMP-II riboswitch) had lower identity in nonpandemic strains (light brown and brown colors; Figure 2, Table S2) compared to the reference strain, while the two RNA-OUT sRNAs, svpa1401.1 and svpa1453.1, were only identified in pandemic strains (black color, Figure 2 and Table S2). To investigate if both RNA-OUT were exclusively present in all pandemic strains, or if it was a particular phenomenon observed only in Chilean strains, we performed more detailed analysis using more than 50 genomes of *V. parahaemolyticus* strains isolated worldwide, available in the National Center for Biotechnology Information (NCBI) database (Table S3). We observed that the presence of both RNA-OUT was also associated with other non-Chilean pandemic strains carrying VPaI-7, which also codes T3SS2. These strains were also ST3 according to MLST, as well as positive for *tdh*, *toxRS*, and *VPaI-7* genes (Table S3). Other strains carrying RNA-OUT were prepandemic strains AQ3810 [24] and BB220P [25], and strains 3259 and EKP-008 (Table S3).

### 2.3. Genomic Context of sRNAs Highly Conserved among Strains Differing in Pandemic Origin

Since we observed that some sRNAs were 100% conserved between nonpandemic strains and reference strain RIMD2210633 (Table S2), we investigated if these sRNAs were acquired by horizontal gene transfer (HGT) from pandemic to nonpandemic strains or vice versa. The criteria were first to determine if sRNAs and their genomic context were fully conserved between nonpandemic strains and the reference strain, and then to search for inverted repeat sequences, integrases, or transposases in the genomic context that could indicate HGT events. Analysis of the immediate genomic context (initially including 500 bp upstream plus 500 bp downstream) of 100% conserved sRNAs (svpa113.1, svpa117.1, svpa1079.1, svpa1370.1, and svpa1729.1) between RIMD2210633 and nonpandemic strains (PMA12.14, PMA14.14, and PMA2.15), showed that there were single-nucleotide variants (SNV) in the flanking sequences, and only the sRNA sequence was 100% conserved between pandemic and nonpandemic strains. The percentage of identity calculated for the genomic context of nonpandemic strains compared to the RIMD2210633 strain was about 98–99% (Table S4), as was expected for bacteria belonging to the same species. PMA37.5 was used as a Chilean pandemic strain control. As was expected, sRNAs and genomic context were fully conserved compared to the pandemic reference strain (Table S4).

### 2.4. Identification of Target Gene Regulated by svpa1401.1 and svpa1453.1

**Figure 3:**
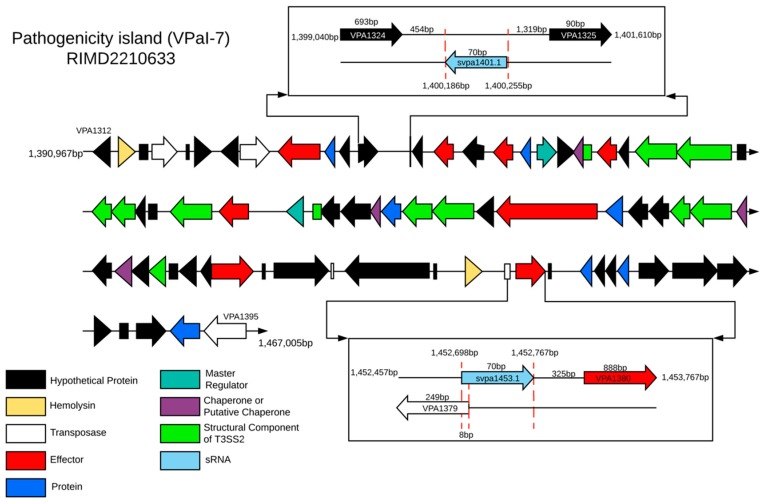
Identification of the target gene regulated by svpa1401.1 and svpa1453.1, and their genomic context visualized in the genome of reference strain RIMD2210633. VPaI-7 defined by Makino and collaborators [28], and Xu and collaborators [29].

Our analysis showed that both RNA-OUT, svpa1401.1 and svpa1453.1, were coded into the VPaI-7 of chromosome II of pandemic strains (Figure 3). Bioinformatics analysis with CopraRNA [26] and TargetRNA2 [27] showed that both sRNAs interact with the transposase VPA1379 coded in the same island (Figure 3), outside of T3SS2. However, svpa1453.1, coded in the opposite strain of VPA1379, was fully complementary with the target gene (acting in *cis*), while svpa1401.1 was coded away from its target (Figure 3), and probably acts in *trans* through binding to the Hfq protein.

### 2.5. Adjustment to the Model of Antisense Regulation of IS10 Expression

**Figure 4:**
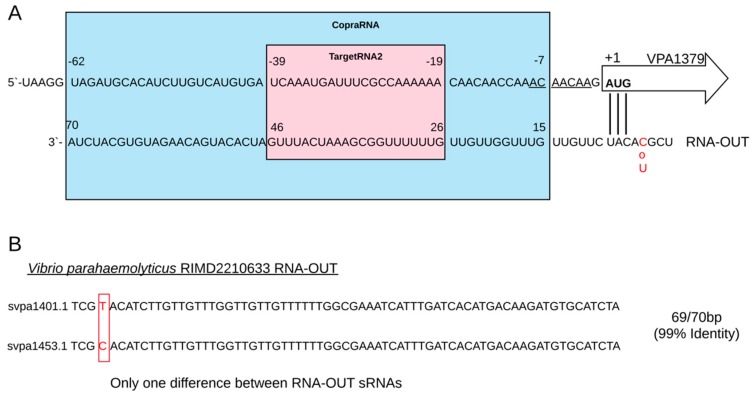
Complementarity of RNA-OUT sRNA and 5′-TIR of transposase VPA1379 mRNA of *V. parahaemolyticus* reference strain RIMD2210633. (**A**) Sequences of RNA-OUT and VPA mRNA shown as linear structures (red characters indicate the difference between both RNA-OUT). Consensus sequence of Hfq binding 5′-AACAACAA-3′ in mRNA is underlined. Start codon AUG is marked in bold. (**B**) Sequences of both RNA-OUT, and only one difference between them when sequences are aligned (red characters in red frame)**.** To review the proposed secondary structures for pairing with or without Hfq, please see Figure 3B in Reference [5].

The accepted model for the IS10 post-transcriptional regulation is based on antisense RNA complementarity to the 5′-translational initiation region (TIR) of the transposase. Thus, translation is impaired by a steric occlusion of the ribosome binding site. Our alignment by BLASTp showed that VPA1379 was similar to a putative IS10 transposase identified in *Salmonella enterica subsp. enterica serovar Typhi*, sharing 85% identity. Additionally, our results showed that both RNA-OUT found in pandemic strains of *V. parahaemolyticus* have around 87.14–90% of identity (svpa1401.1: 87.14–88.57%; svpa1453.1; 88.57–90.0%), while VPA1379 have 85.53% of identity compared to *Escherichia coli* (taxid:562). We also identified that RNA-OUT svpa1453.1 was perfectly complementary to the VPA1379 mRNA in the 5′-TIR (Figure 4A), while RNA-OUT svpa1401.1 only had one difference compared to svpa1453.1, but this nucleotide paired outside of the 5′-TIR (Figure 4B). Our results suggest that pairing between each RNA-OUT and VPA1379 would prevent transposase translation by steric occlusion of the ribosome binding site. We also identified a binding motif to Hfq in the 5′-TIR of VPA1379 (5′-AACAACAA-3′) and U-rich regions in both RNA-OUT, suggesting that antisense pairing could be facilitated by binding to the Hfq protein, as was suggested with *Escherichia coli* [5]. Unexpectedly, we could not identify a clear Shine–Dalgarno consensus sequence in the 5′-TIR of VPA1379 (Figure 4A).

### 2.6. VPA1379 mRNA and RNA-OUT svpa1401.1 Expression during in vitro Infection

**Figure 5:**
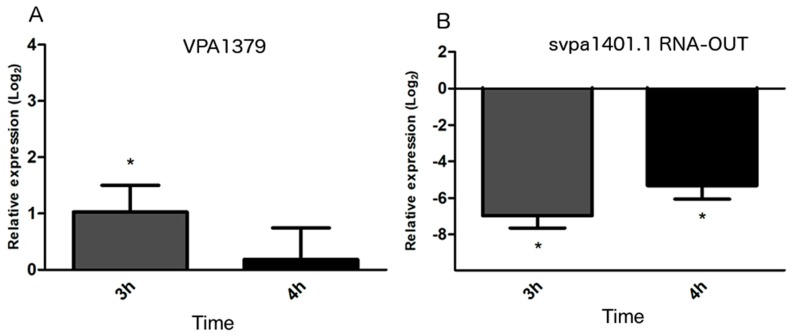
Transcription level of VPA1379 mRNA and svpa1401.1 RNA-OUT during infection of Caco-2 cells. (**A**) VPA1379 mRNA relative expression in 3 and 4hpi; (**B**) svpa1401 RNA-OUT relative expression in 3 and 4hpi. Significant differences of each time compared with 0 hpi (asterisk marks) were considered as *p* < 0.05 using REST software analysis and statistical comparison.

The VpKX strain exhibited a significant increase of VPA1379 relative expression at 3 h postinfection (hpi) of Caco-2 cells, while the observed increase of VPA1379 mRNA at 4hpi compared to 0hpi was not significant (Figure 5A). On the contrary, the relative expression of RNA-OUT svpa1401.1 was significantly decreased at 3hpi and 4hpi comparing to 0hpi (Figure 5B). For both genes, the relative expression was calculated according to the expression of the 23S used as the normalizer gene.

## 3. Discussion

Current high-throughput sequencing technology provides a means for the discovery of novel genes [30] including sRNAs. Nonetheless, there is little knowledge on the extent of sequence conservation of sRNAs in bacteria [31]. The evolution of some sRNA sequences seems to be rapid and could result in limited similarity of sRNAs, even between closely related species [17]. Despite this, there are classical conserved sRNAs in bacteria such as 6S RNA, 4.5S rRNA, RNase P RNA, and tmRNA. SRNA conservation seems to be strongly related to function. For example, there is a large number of non-conserved sRNAs in *Pseudomonas*, reflecting the metabolic diversification and niche specialization of this species, while most highly conserved sRNAs are associated with conserved regulatory networks, such as iron metabolism and quorum-sensing regulation [31]. Thus, we expected that sRNAs associated with conserved networks in *Vibrio* species would fulfill similar functions. However, comparison of sRNAs between *V. parahaemolyticus* and the two other *Vibrio* species showed that only three sRNAs, RNaseP_bact_a, RyhB, and Spot42, were conserved between the three species with 90% identity (Figure 1), RyhB and Spot42 being the unique sRNAs experimentally studied in *V. parahaemolyticus*. Pairwise comparisons suggested that *V. parahaemolyticus* and *V. vulnificus* share more similar regulatory functions (nine sRNAs) than *V. parahaemolyticus* and *V. cholerae* (only one sRNA). Four of the nine sRNAs, highly conserved in *V. parahaemolyticus* and *V. vulnificus* (RNaseP_bact_a, mini-ykkC, S15, P26), are also conserved in bacteria because they have essential functions in the regulation of key common and vital processes in different species.

On the other hand, there are sRNAs with related functions but lack of sequence similarity. This observation has led to looking for other ways to investigate the functional conservation of sRNAs, including analysis of gene context [17]. Considering this information, we continued our exploratory study comparing sRNAs between *V. parahaemolyticus* strains by considering their genomic context. We noticed that the 43 sRNAs described for *V. parahaemolyticus* in BSRD (Table S1) were found in the twenty Chilean strains of *V. parahaemolyticus* with very few exceptions (Figure 2). Nine sRNAs showed 100% identity between pandemic and nonpandemic strains, including Alpha_RBS, mini-ykkC, ffs (also called 4.5 rRNA), Cyclic di-GMP-II riboswitch, Lysine riboswitch, Purine riboswitches, S15, P26, and TPP riboswitch. They are related to several conserved processes, including translation, sensing of amino acids (Lysine riboswitch, Purine riboswitches) and vitamins (TPP or vitamin B1-riboswitch). As was expected, five classical sRNAs were highly conserved in bacteria: 6S RNA (svpa2734.1), 4.5S rRNA (svpa996.1), RNase P RNA (svpa452.1), RNaseP_bact_a, and tmRNA were also highly conserved in *V. parahaemolyticus*, although some strains showed slight differences in sequences. As an exception, clinical strain PMC58.5 showed only 78.4% identity in 6S RNA compared to the reference strain (Figure 2, Table S2). Since this sRNA inhibits transcription by binding directly to the housekeeping holoenzyme form of RNA polymerase [32], PMC58.5 may have differences in its transcriptional activity compared to other pandemic strains.

Interestingly, analysis of the genomic context of sRNAs 100% conserved among pandemic and nonpandemic strains showed that only the sRNA sequences but not their immediate contexts were fully conserved (Tables S2 and S4). This suggests that sRNAs are fully conserved in strains differing in pandemic origin due to the importance of their function, probably related to the viability or fitness of the strain, and not because they were acquired by HGT. On the contrary, the TPP riboswitch, Cyclic-di-GMP-II riboswitch, and RyeB showed sequence differences between pandemic and nonpandemic strains, while the two RNA-OUT sRNAs were exclusively present in pandemic strains. Our subsequent computational analysis confirmed that both RNA-OUT were found in the genomes of strains of *V. parahaemolyticus* containing VPaI-7 and other genes such as *tdh* and T3SS2, indicating a pandemic or prepandemic origin. In fact, a plausible explanation of the pandemic O3:K6 strain emergence suggests that an ancestral strain possessing the O3:K6 serotype recruited a *tdh* containing island, such as VPaI-7 (see Figure 1 in [24]).

Additionally, we noticed that both RNA-OUT were encoded by VPaI-7, which also contains both *tdh* genes and T3SS2, the classical virulence factors described for this species [15], in addition to five transposase genes. The presence of transposase genes in mobile elements, such as phages, plasmids, and genomic islands, has much evolutionary importance because it facilitates the dissemination of DNA transposons and, consequently, increases the diversity of bacterial species [33]. However, since these mobile segments can often encode viral genes, catabolic genes, virulence factors, and antibiotic resistance genes, the favored dissemination between bacterial species also represents a public health hazard, and consequently, the knowledge of transposition regulation in human pathogenic bacteria is a relevant issue.

The maintenance of a transposable element depends on the balance between the duplication of the element and the possible negative effects that DNA rearrangements can cause to the host [34]. Thus, it is crucial to the host to possess mechanisms that allow diminishing or avoiding the expression of transposases [34]. Resuming the mechanisms to control transposition activities depends on intrinsic mechanisms of IS10/Tn10 and on the interplay between IS10 and host. Usually, the frequency of IS10 transposition is low because transcription is infrequent due to the weakness of the promoter, and translation is also inefficient due to the absence of a clear Shine–Dalgarno sequence [35], as we also evidenced in the 5′-TIR of VPA1379 (Figure 4A). Additionally, another regulatory mechanism of IS10 occurs through adenine methylation [35], while there is also a mechanism of antisense regulation mediated by sRNA [5]. The sRNA responsible for the mechanism of antisense regulation of the Tn10/IS10 system is called RNA-OUT, and it has been extensively described in *E. coli* [5]. In this model of antisense regulation, RNA-OUT is complementary to the 5′ end of the transposase mRNA (RNA-IN), limiting transposase translation through the sequestration of the ribosome binding site [36].

According to our analysis, both RNA-OUT of *V. parahaemolyticus*, svpa1453.1 and svpa1401.1, would interact with transposase VPA1379, which is also encoded by VPaI-7. Our results also showed that svpa1453.1 was coded in the opposite strand, suggesting there is a *cis*-acting sRNA with perfect complementarity to the target, while svpa1401.1 being coded away from VPA1379 was probably acting in *trans* (Figure 3), depending on Hfq binding. In fact, we identified an Hfq-binding motif in the 5′-TIR of VPA1379 (5′-AACAACAA-3′) and U-rich regions in both RNA-OUT, suggesting that the antisense pairing mechanism in *V. parahaemolyticus* could also be facilitated by binding to the Hfq protein, as was shown with *E. coli* [5]. It has been demonstrated that the frequency of transposition in *E. coli* is greatly increased in *hfq* mutant strains, implicating Hfq as a potent negative regulator of transposition through RNA-OUT. Despite RNA-OUT being an antisense RNA, binding to Hfq is necessary for the correct pairing with the mRNA of transposase and, then, to the regulation of IS10 transposition [37].

Additionally, the regulation of transposition has also been linked to cell-stress response pathways [38]. In fact, it is known that the IS10 transposition increases the fitness of *E. coli* [39] and, in the presence of UV radiation, IS10 transposition is induced by the involvement of the SOS stress response, probably to increase bacterial survival while facing adverse environmental conditions [40]. In this same context, pathogenic bacteria must face diverse host environments during the infective process, which trigger the expression of several genes in response to these specific stress conditions [37]. So, if IS10 transposition increased in response to a stress condition, it could be expected that IS10 transposition would increase during infection. Even more so, since RNA-OUT and transposase VPA1379 were coded in VPaI-7, where all classical virulence determinants of *V. parahaemolyticus* are encoded and expressed during infective process, we hypothesized that transposase, RNA-OUT, or both could increase their expression in the pandemic strain VpKX during infection. Effectively, we observed that the relative expression of VPA1379 was significantly increased at 3hpi, while RNA-OUT was decreased at the same time. To contrast our results, we downloaded the RNA-Seq free available data of Livny [21] and we analyzed the gene expression of transposase/RNA-OUT in the infection of a rabbit ileal ceca condition compared with a laboratory isolation condition. We observed that transposase VPA1379 was upregulated (2.4-fold), but both RNA-OUT svpa1401.1 and svpa1453.1 were also upregulated (2.7-fold and 2.3-fold, respectively), in the infection conditions [21]. Based on both studies, we propose that VPA1379 transcription increases during infection of *V. parahaemolyticus* probably because IS10 transposition is also favored under stress conditions in this species.

On the other hand, analysis of RNA-OUT expression levels showed that results obtained in both studies were the opposite. In our study, sRNA decreased when the mRNA level of transposase increased, while the results of Livny and collaborators [21] showed that the expression of both RNA-OUT was upregulated when transposase was also upregulated. We are conscious that both techniques and the evaluated conditions in both studies were not the same and, consequently, results were not directly comparable. However, regulation by RNA-OUT occurs at the post-transcriptional level [5], and evidently this antisense mechanism would not occur under in vitro infection, due to the absence of RNA-OUT. On the other hand, the upregulation of RNA-OUT expression during rabbit infection does not necessarily indicate that the antisense mechanism would be exerted. Under adverse conditions, several sRNAs involved in the regulation of different stress pathways increase their expression. When sRNAs are overexpressed, some of them compete with endogenous sRNAs for binding to Hfq, limiting the availability of this protein and, consequently, limiting the function of many *trans*-sRNAs, but also in this antisense RNA-OUT. Since the frequency of transposition is greatly increased in *hfq* mutant strains of *E. coli*, an increase of RNA-OUT has no effect in transposase regulation in the absence of available Hfq [37].

The obtained results in this study and additional analysis of available free data showed and confirmed, respectively, that IS10 transcription increases in pandemic strains of *V. parahaemolyticus* under infection conditions. Although the basal transcription level of transposase is very low due to the weakness of the IS10 promoter, we propose that, under adverse conditions, pandemic strains increase transcription of IS10, probably to favor transposition and, consequently, genomic diversity and their fitness. However, whether IS10 transposition means an advantage for the fitness of pandemic strains of *V. parahaemolyticus* compared with nonpandemic strains, which lack an RNA-OUT/transposase system, is a matter for future studies.

## 4. Materials and Methods

### 4.1. Strains

Twenty strains of clinical and environmental origin (Table 1) were grown in Luria Bertani (LB) broth with 3% NaCl and tested for *tlh*, *tdh*, or *trh* by multiplex PCR (mPCR), plated on CHROMagar Vibrio (CHROMagar Microbiology, Paris, France), and analyzed by direct genome restriction enzyme analysis (DGREA) to distinguish pandemic from nonpandemic strains [41]. Pandemic strain RIMD2210633 of *V. parahaemolyticus* (also called VpKX) was obtained from the Research Institute for Microbial Diseases, Osaka University, Osaka, Japan [42], and donated to this study by Romilio Espejo.

### 4.2. DNA Extraction, Sequencing, and Genome Data

*V. parahaemolyticus* strains were analyzed and sequenced as previously described [15,22]. Briefly, bacteria were grown overnight in LB broth, and DNA was extracted with a Wizard Genomic DNA Purification Kit (Promega Corporation, Madison, WI, USA). DNA sequencing of VpKX, ATC210.98, ATC220.98, PMC48.4, PMC58.5, PMA37.5, PMA109.5, PMC14.7, and PMC58.7 strains was performed in Ion Torrent for single-end using 100 bp chemistry libraries (Life Technologies, Foster City, CA, USA), and in 454 FLX+ for mate paired-end libraries with a 3000 bp span (Roche, Indianapolis, IN, USA) (Table 1). Preparation and sequencing of libraries were performed following the respective manufacturer’s instructions [22].

DNA sequencing of *V. parahaemolyticus* strains PMA1.15, PMA2.15, and PMA3.15 was performed in Ion Torrent PGM for single-end using 100 bp chemistry libraries (Life Technologies, Foster City, CA, USA). DNA sequencing of *V. parahaemolyticus* strains PMC53.7, PMC54.13, PMA14.14, PMA11.14, PMA12.14, PMA32.14, PMA21.14, PMA31.14, and PMC81.13 was performed in Illumina MiSeq platform (Table 1). Paired-end library preparation and sequencing were performed following the respective manufacturer’s instructions for Illumina TruSeq DNA protocol [15].

### 4.3. SRNA Sequence Comparison between Human Pathogenic Vibrio Strains

sRNA sequences of *Vibrio* species were downloaded from the BSRD database [43] (164 sequences of *V. cholerae*, 43 of *V. parahaemolyticus,* and 40 of *V. vulnificus*). sRNA sequences of each strain were concatenated into a single FASTA file; these sequences were clustered by 102 VSEARCH v.2.7 [44] with the cluster_fast module, using 90%, 95%, and 100% nucleotide identity. Identity for comparison was defined as the edit distance excluding terminal gaps. Sequences of any length were compared using only the plus strand. Results were tabulated with a custom script for further inspection.

### 4.4. Conservation and Distribution of sRNA in V. parahaemolyticus Strains

The analysis of sequence-identity percentage and distribution of sRNA in *V. parahaemolyticus* strains (presence/absence) was performed by MUMmer v.3.0 [45] using the assemblies of each genome compared to a reference file containing the sequence of 43 known sRNAs described for pandemic *V. parahaemolyticus* RIMD2210633 [28] in the BSRD database [43]. The genomic context of fully conserved sRNAs between pandemic and nonpandemic strains was studied using coding RIMD2210633 genome as reference, reconstructing the sequence of each sRNA and its genomic context for each strain. The filtered reads were corrected and aligned with SMALT v0.7.4. SNVs were calculated for each strain using FreeBayes v1.1 [46]. Noncover positions were calculated with Genomecov of BEDTools v2.26 [47]. Using the information of noncover positions and SNVs, the sRNA sequence was rebuilt for each strain using a Python script. Visualization of the possible genome context of sRNAs on reads alignment against reference RIMD2210633 was conducted using Artemis Comparison Tool (ACT), release v13.0.0 [48]. Additionally, 50 genomes of *V. parahaemolyticus* strains were obtained from the NCBI database (Table S3). They were classified as pandemic or nonpandemic strains according to MLST-sequence type 3, the absence of *trh*, and the presence of *orf8*, *toxRS*, *tdh,* and VPaI-7 [24,29]. The presence of the genes and sRNA of svpa1401.1 and svpa1453.1 was identified using BLAST comparison [49].

### 4.5. Infection Assay

*V. parahaemolyticus* strain VpKX was used to infect Caco-2 cells. Cells were grown in Dulbecco’s Modified Eagle’s medium (DMEM; Sigma-Aldrich, St. Louis, MO, USA) supplemented with 10% fetal bovine serum (FBS; Grand Island, Gibco, NY, USA) plus 1% penicillin–streptomycin solution (Grand Island, Gibco, NY, USA), and were maintained in a 75 mL flask at 37 °C under 5% CO_2_ in a humidified incubator until confluence. For infections, cells were seeded in a 6-well plate (5 × 10^6^ cells/well) and incubated for 48 h in DMEM– 10% FBS. Growth medium was removed from 80–90% of confluent Caco-2 monolayers and washed 3 times with phosphate-buffered saline. A culture in exponential phase (OD_600_ = 0.6) of *V. parahaemolyticus* VpKX strain in LB 3% NaCl was centrifuged and, subsequently, a bacterial suspension was prepared in DMEM without phenol red or antibiotics at a multiplicity of infection (M.O.I = 10), after standardization. At the onset of infection, cells were centrifuged at 250× *g* for 4 min to synchronize the contact of the bacteria to the cell and incubated for 4 h at 37 °C and 5% CO_2._

### 4.6. RNA Extraction, cDNA Synthesis, and Real-Time Quantitative PCR (RT-qPCR)

Cells were collected for RNA extraction at 0, 3, and 4 hpi. RNA was isolated using E.Z.N.A Total RNA kit (Omega Bio-Tek, Norcross, GA, USA) according to the manufacturer’s instructions and quantified using an Infinite M200pro spectrophotometer. Complementary DNA (cDNA) was synthesized through random hexamer-primed reactions using ImProm-II Reverse Transcriptase (Promega, WI, USA). RT-qPCR was performed using a LightCycler 96 with FastStart Essential DNA Green Master (Roche, Indianapolis, IN, USA) following the manufacturer’s instructions. Pairs of primers used for conventional PCR and RT-qPCR were: Transposase (TranspF: 5′-CTCTACCAATTCTGCCCTGAAC-3′ and TranspR: 5′-CCAAGTTTGGTGAGCGTAAGA-3′); RNA-OUT 1401.1 (svpa1401.1F: 5′-GTACATCTTGTTGTTTGG-3′ and svpa1401.1R: 5′-ATGTGATCAAATGATTTCG-3′) and 23S (23SF: 5′-GTCCCGTAGTTGACGACGTG-3′ and 23SR: 5′-ACGCAGTCACAGGACAAAGCC-3′). 23S was used as the reference gene [50].

The thermal cycling profile was performed at 95 °C for 5 min, followed by 40 cycles of 95 °C for 20 s, 60 °C for 20 s, and 72 °C for 10 s. A melting curve performed at the end of the amplification was undertaken to confirm that there was only a single product amplified in each reaction. Each RT-qPCR reaction was conducted in triplicate. Threshold cycle (Ct) and melting-curve analysis were performed using LightCycler 96 software (Roche, Indianapolis, IN, USA) v1.1.0.1320. The expression level of each gene was calculated using the 2^−ΔΔ*C*t^ method after normalization to 23S rRNA with REST 2009 software (Qiagen, Hilden, Germany) [51].

### 4.7. Sequence-Based Analysis of RNA-OUT Target and Genomic-Context Visualization

Determination of the putative target of both RNA-OUT in *V. parahaemolyticus* was carried out by CopraRNA [26] and TargetRNA2 [27]. For target identification by CopraRNA, which predicts the possible target of action based on RNA–RNA interaction, it was necessary to search for homologous sequences in databases with GLASSgo [52], which allowed the identification of similar sequences in different organisms for RNA-OUT. We used CopraRNA with at least 3 homologous sRNA sequences from 3 different organisms: *Escherichia coli* O157: H7, *Salmonella enterica* subsp. *enterica* serovar Choleraesuis str. SC-B67, and *Pseudoalteromonas atlantica* T6c. TargetRNA2 identified possible candidates by base-pairing in possible targets, especially by secondary structure and hybridization energy. To determine the exact location of both sRNAs and the differences between them, the search of genomic context was carried out by BLAST [49] and visualized using Artemis [48].

## 5. Conclusions

Although high conservation of several sRNAs was observed across pandemic and nonpandemic *V. parahaemolyticus* strains, two sRNAs coding for RNA-OUT were exclusively present in pandemic strains. Both of these RNA-OUT encoded in VPaI-7 and would interact with the VPA1379 transposase according to the model of pairing IS10-encoded antisense RNAs. Since the mRNA level of VPA1379 increased during the infective process, and it was demonstrated that the increase of IS10 transposition improves the fitness of bacterial species, we suggest that the regulation of VPA1379 by RNA-OUT could be advantageous to the fitness of *V. parahaemolyticus* pandemic strains under stress conditions.

## Figures and Tables

**Table 1:** Chilean *V. parahaemolyticus* strains analyzed in this study.

Strain	Isolate Year	Origin	Source	DGREA Classification	Accession Number and/or SRA	Sequencing Platform	Reference
ATC210.98	1998	Antofagasta, Chile	Stool	Pandemic	LFUN00000000 SRR1301223	Ion Torrent	[22]
ATC220.98	1998	Antofagasta, Chile	Stool	Pandemic	LFUQ00000000 SRR1293142	Ion Torrent	[22]
PMC48.4	2004	Puerto Montt, Chile	Stool	Pandemic	LFUP00000000 SRR1292943	Ion Torrent	[22]
PMC58.5	2005	Puerto Montt, Chile	Stool	Pandemic	LFUJ00000000 SRR1292941	Ion Torrent	[22]
PMA37.5	2005	Puerto Montt, Chile	Mussels	Pandemic	LFUL00000000 SRR1301224	Ion Torrent	[22]
PMA109.5	2005	Puerto Montt, Chile	Mussels	Pandemic	LFUK00000000 SRR1293140	Ion Torrent	[22]
PMC14.7	2007	Puerto Montt, Chile	Stool	Pandemic	LFUO00000000 SRR1293138	Ion Torrent	[22]
PMC53.7	2007	Puerto Montt, Chile	Stool	Nonpandemic	MKQF00000000	Illumina MiSeq	[15]
PMC58.7	2007	Puerto Montt, Chile	Stool	Pandemic	LFUM00000000 SRR1292939	Ion Torrent	[22]
PMC54.13	2013	Puerto Montt, Chile	Stool	Nonpandemic	MKQX00000000	Illumina MiSeq	[15]
PMC81.13	2013	Puerto Montt, Chile	Stool	Pandemic	SRR3002506	Illumina MiSeq	[23]
PMA11.14	2014	Puerto Montt, Chile	Mussels	Nonpandemic	MKQY00000000	Illumina MiSeq	This study
PMA12.14	2014	Puerto Montt, Chile	Mussels	Nonpandemic	MKQZ00000000	Illumina MiSeq	This study
PMA14.14	2014	Puerto Montt, Chile	Mussels	Nonpandemic	MKRA00000000	Illumina MiSeq	[15]
PMA21.14	2014	Puerto Montt, Chile	Mussels	Nonpandemic	MKRB00000000	Illumina MiSeq	This study
PMA31.14	2014	Puerto Montt, Chile	Mussels	Nonpandemic	MKRC00000000	Illumina MiSeq	This study
PMA32.14	2014	Puerto Montt, Chile	Mussels	Nonpandemic	MKRD00000000	Illumina MiSeq	This study
PMA1.15	2015	Puerto Montt, Chile	Mussels	Nonpandemic	MKQV00000000	Ion Torrent	[15]
PMA2.15	2015	Puerto Montt, Chile	Mussels	Nonpandemic	MKQT00000000	Ion Torrent	[15]
PMA3.15	2015	Puerto Montt, Chile	Mussels	Nonpandemic	MKQU00000000	Ion Torrent	[15]

## Supplementary Materials

Supplementary materials can be found at https://www.mdpi.com/1422-0067/20/11/2827/s1.

## References

[1] Zhang Y., Qiu Y., Tan Y., Guo Z., Yang R., Zhou D. (2012). Transcriptional regulation of opaR, qrr2-4 and aphA by the master quorum-sensing regulator opaR in *Vibrio parahaemolyticus*. PLoS ONE.

[2] Dutta T., Srivastava S. (2018). Small RNA-mediated regulation in bacteria: A growing palette of diverse mechanisms. Gene.

[3] Papenfort K., Vanderpool C.K. (2015). Target activation by regulatory RNAs in bacteria. FEMS Microbiol. Rev..

[4] Perez-Reytor D., Plaza N., Espejo R.T., Navarrete P., Bastias R., Garcia K. (2017). Role of non-coding regulatory RNA in the virulence of human pathogenic *Vibrios*. Front. Microbiol..

[5] Ross J.A., Ellis M.J., Hossain S., Haniford D.B. (2013). Hfq restructures RNA-IN and RNA-OUT and facilitates antisense pairing in the Tn 10/IS10 system. RNA.

[6] Heidelberg J.F., Heidelberg K.B., Colwell R.R. (2002). Seasonality of chesapeake bay bacterioplankton species. Appl. Environ. Microbiol..

[7] Huang Z., Liu Z., Shao Z. (2017). The pelagic bacterium *Paraphotobacterium marinum* has the smallest complete genome within the family vibrionaceae. Front. Microbiol..

[8] Thompson F.L., Iida T., Swings J. (2004). Biodiveristy of *Vibrios*. Microbiol. Mol. Biol. Rev..

[9] Plaza N., Castillo D., Pérez-Reytor D., Higuera G., García K., Bastías R. (2018). Bacteriophages in the control of pathogenic *Vibrios*. Electron. J. Biotechnol..

[10] Song T., Wai S.N. (2009). A novel sRNA that modulates virulence and environmental fitness of *Vibrio cholerae*. RNA Biol..

[11] Song T., Sabharwal D., Gurung J.M., Cheng A.T., Sjöström A.E., Yildiz F.H., Uhlin B.E., Wai S.N. (2014). *Vibrio cholerae* utilizes direct sRNA regulation in expression of a biofilm matrix protein. PLoS ONE.

[12] Richard A.L., Withey J.H., Beyhan S., Yildiz F., DiRita V.J. (2010). The *Vibrio cholerae* virulence regulatory cascade controls glucose uptake through activation of TarA, a small regulatory RNA. Mol. Microbiol..

[13] Bradley E.S., Bodi K., Ismail A.M., Camilli A. (2011). A genome-wide approach to discovery of small RNAs involved in regulation of virulence in *Vibrio cholerae*. PLoS Pathog..

[14] García K., Bastías R., Higuera G., Torres R., Mellado A., Uribe P., Espejo R.T. (2013). Rise and fall of pandemic *Vibrio parahaemolyticus* serotype O3: K6 in southern Chile. Environ. Microbiol..

[15] Castillo D., Pérez-Reytor D., Plaza N., Ramírez-Araya S., Blondel C.J., Corsini G., Bastías R., Loyola D.E., Jaña V., Pavez L. (2018). Exploring the genomic traits of non-toxigenic *Vibrio parahaemolyticus* strains isolated in southern Chile. Front. Microbiol..

[16] Paranjpye R.N., Myers M.S., Yount E.C., Thompson J.L. (2013). Zebrafish as a model for *Vibrio parahaemolyticus* virulence. Microbiol. (UK).

[17] Gottesman S., Storz G. (2011). Bacterial small RNA regulators: Versatile roles and rapidly bacterial small RNA regulators. Cold Spring Harb. Perspect. Biol..

[18] Tanabe T., Funahashi T., Nakao H., Maki J., Yamamoto S. (2013). The *Vibrio parahaemolyticus* small RNA ryhb promotes production of the siderophore vibrioferrin by stabilizing the polycistronic mRNA. J. Bacteriol..

[19] Tanabe T., Miyamoto K., Tsujibo H., Yamamoto S., Funahashi T. (2015). The small RNA Spot 42 regulates the expression of the type III secretion system 1 (T3SS1) chaperone protein VP1682 in *Vibrio parahaemolyticus*. FEMS Microbiol. Lett..

[20] García K., Yáñez C., Plaza N., Peña F., Sepúlveda P., Pérez-Reytor D., Espejo R.T. (2017). Gene expression of *Vibrio parahaemolyticus* growing in laboratory isolation conditions compared to those common in its natural ocean environment. BMC Microbiol..

[21] Livny J., Zhou X., Mandlik A., Hubbard T., Davis B.M., Waldor M.K. (2014). Comparative RNA-Seq based dissection of the regulatory networks and environmental stimuli underlying *Vibrio parahaemolyticus* gene expression during infection. Nucleic Acids Res..

[22] Loyola D.E., Navarro C., Uribe P., García K., Mella C., Díaz D., Valdes N., Martínez-Urtaza J., Espejo R.T. (2015). Genome diversification within a clonal population of pandemic *Vibrio parahaemolyticus* seems to depend on the life circumstances of each individual bacteria. BMC Genomics.

[23] Loyola D.E., Yañez C., Plaza N., Garcia K., Espejo R.T. (2016). Genealogy of the Genome Components in the Highly Homogeneous Pandemic *Vibrio parahaemolyticus* Population. J. Phylogenetics Evol. Biol..

[24] Espejo R.T., García K., Plaza N. (2017). Insight into the origin and evolution of the *Vibrio parahaemolyticus* pandemic strain. Front. Microbiol..

[25] Bhowmik S.K. (2019). Study of Five Complete Genomes of *Vibrio parahaemolyticus*: Focusing Non-Pandemic to Pandemic Development. Int. J. Sci. Res..

[26] Wright P.R., Georg J., Mann M., Sorescu D.A., Richter A.S., Lott S., Kleinkauf R., Hess W.R., Backofen R. (2014). CopraRNA and IntaRNA: Predicting small RNA targets, networks and interaction domains. Nucleic Acids Res..

[27] Kery M.B., Feldman M., Livny J., Tjaden B. (2014). TargetRNA2: Identifying targets of small regulatory RNAs in bacteria. Nucleic Acids Res..

[28] Makino K., Oshima K., Kurokawa K., Yokoyama K., Uda T., Tagomori K., Iijima Y., Najima M., Nakano M., Yamashita A. (2003). Genome sequence of *Vibrio parahaemolyticus*: A pathogenic mechanism distinct from that of *V cholerae*. Lancet.

[29] Xu F., Gonzalez-Escalona N., Drees K.P., Sebra R.P., Cooper V.S., Jones S.H., Whistler C.A. (2017). Parallel evolution of two clades of an Atlantic-Endemic Pathogenic Lineage of *Vibrio parahaemolyticus* by independent acquisition of related pathogenicity islands. Appl. Environ. Microbiol..

[30] Nydam S.D., Shah D.H., Call D.R. (2014). Transcriptome analysis of *Vibrio parahaemolyticus* in type III secretion system 1 inducing conditions. Front. Cell. Infect. Microbiol..

[31] Gómez-Lozano M., Marvig R.L., Molina-Santiago C., Tribelli P.M., Ramos J.L., Molin S. (2015). Diversity of small RNAs expressed in *Pseudomonas* species. Environ. Microbiol. Rep..

[32] Hnilicová J., Jirát Matějčková J., Šiková M., Pospíšil J., Halada P., Pánek J., Krásný L. (2014). Ms1, a novel sRNA interacting with the RNA polymerase core in mycobacteria. Nucleic Acids Res..

[33] Siguier P., Gourbeyre E., Chandler M. (2014). Bacterial insertion sequences: Their genomic impact and diversity. FEMS Microbiol. Rev..

[34] Craig N., Neidhardt F. (1996). Transposition. EcoSal—Escherichia coli and Salmonella: Cellular and Molecular Biology.

[35] Crow J.F., Dove W.F. (2000). Perspectives on Genetics: Anecdotal, Historical, and Critical Commentaries, 1987–1998.

[36] Ma C., Simons R.W. (1990). The IS10 antisense RNA blocks ribosome binding at the transposase translation initiation site. EMBO J..

[37] Ross J.A., Wardle S.J., Haniford D.B. (2010). Tn10/IS10 transposition is downregulated at the level of transposase expression by the RNA-binding protein Hfq. Mol. Microbiol..

[38] Twiss E., Coros A.M., Tavakoli N.P., Derbyshire K.M. (2005). Transposition is modulated by a diverse set of host factors in *Escherichia coli* and is stimulated by nutritional stress. Mol. Microbiol..

[39] Chao L., Vargas C., Spear B.B., Cox E.C. (1983). Transposable elements as mutator genes in evolution. Nature.

[40] Eichenbaum Z., Livneh Z. (1998). UV light induces IS10 transposition in *Escherichia coli*. Genetics.

[41] Fuenzalida L., Hernández C., Toro J., Rioseco M.L., Romero J., Espejo R.T. (2006). *Vibrio parahaemolyticus* in shellfish and clinical samples during two large epidemics of diarrhoea in southern Chile. Environ. Microbiol..

[42] García K., Torres R., Uribe P., Hernández C., Rioseco M.L., Romero J., Espejo R.T. (2009). Dynamics of clinical and environmental *Vibrio parahaemolyticus* strains during seafood-related summer diarrhea outbreaks in southern Chile. Appl. Environ. Microbiol..

[43] Li L., Huang D., Cheung M.K., Nong W., Huang Q., Kwan H.S. (2013). BSRD: A repository for bacterial small regulatory RNA. Nucleic Acids Res..

[44] Rognes T., Flouri T., Nichols B., Quince C., Mahé F. (2016). VSEARCH: A versatile open source tool for metagenomics. Peer J..

[45] Kurtz S., Phillippy A., Delcher A.L., Smoot M., Shumway M., Antonescu C., Salzberg S.L., Kurtz S., Phillippy A., Delcher A.L. (2004). Versatile and open software for comparing large genomes. Genome Biol..

[46] Garrison E., Marth G. (2012). Haplotype-based variant detection from short-read sequencing. arXiv.

[47] Quinlan A.R., Hall I.M. (2010). BEDTools: A flexible suite of utilities for comparing genomic features. Bioinformatics.

[48] Carver T., Berriman M., Tivey A., Patel C., Böhme U., Barrell B.G., Parkhill J., Rajandream M.A. (2008). Artemis and ACT: Viewing, annotating and comparing sequences stored in a relational database. Bioinformatics.

[49] Camacho C., Coulouris G., Avagyan V., Ma N., Papadopoulos J., Bealer K., Madden T.L. (2009). BLAST+: Architecture and applications. BMC Bioinform..

[50] Ma Y.J., Sun X.H., Xu X.Y., Zhao Y., Pan Y.J., Hwang C.A., Wu V.C.H. (2015). Investigation of reference genes in *Vibrio parahaemolyticus* for gene expression analysis using quantitative RT-PCR. PLoS ONE.

[51] Pfaffl M.W., Horgan G.W., Dempfle L. (2002). Relative expression software tool (REST) for group-wise comparison and statistical analysis of relative expression results in real-time PCR. Nucleic Acids Res..

[52] Lott S.C., Schäfer R.A., Mann M., Backofen R., Hess W.R., Voß B., Georg J. (2018). GLASSgo—Automated and reliable detection of sRNA homologs from a single input sequence. Front. Genet..

